# Second-Order Vector Mode Propagation in Hollow-Core Antiresonant Fibers

**DOI:** 10.3390/mi10060381

**Published:** 2019-06-07

**Authors:** Lili Li, Limin Xiao

**Affiliations:** 1 Advanced Fiber Devices and Systems Group, Key Laboratory of Micro and Nano Photonic Structures (MoE), Department of Optical Science and Engineering, Fudan University, Shanghai 200433, China; 16210720010@fudan.edu.cn; 2 Key Laboratory for Information Science of Electromagnetic Waves (MoE), Fudan University, Shanghai 200433, China; 3 Shanghai Engineering Research Center of Ultra-Precision Optical Manufacturing, Fudan University, Shanghai 200433, China; 4 Guangdong Provincial Key Laboratory of Optical Information Materials and Technology, South China Academy of Advanced Optoelectronics, South China Normal University, Guangzhou 510006, China

**Keywords:** photonic crystal fiber, hollow-core fiber, suppression of the fundamental mode, second-order mode propagation

## Abstract

Second-order vector modes, possessing doughnut-shaped intensity distribution with unique polarization, are widely utilized in material micromachining, optical tweezers, and high-resolution microscopy. Since the hollow-core fiber can act as a flexible and robust optical waveguide for ultra-short pulse delivery and manipulation, high-order vector modes guided in hollow-core fibers will have huge potential in many advanced applications. We firstly reveal that a second-order vector mode can be well guided in a hollow-core antiresonant fiber with the suppression of the fundamental mode and other second-order vector modes at the red side of transmission band. We interpret our observation through a phase-matched coupling mechanism between core modes and coupled cladding modes. A single second-order vector mode such as TE_01_, TM_01_, or HE_21_ can be guided with low confinement loss at specific wavelengths with appropriate structure parameters. Our proposed hollow-core fibers have a modal engineering function which will open up a new avenue toward the single second-order vector mode propagation and its fiberized applications.

## 1. Introduction

Hollow-core antiresonant fibers (HC-ARFs) attract considerable interest for their simple geometries and extraordinary performances [1,2,3], which are utilized for a wide range of applications such as telecommunications [4], micromachines [5], surgical procedures [6], ultraviolet laser delivery [7], mid-infrared fiber lasers [8], and optofluidic sensors [9]. Additionally, HC-ARFs can also be further exploited as interesting platforms to study complex sensitive processes in gases or microfluidics due to the possibility of filling the hollow core with different gases or liquids, long interaction length, and the ability to suppress unwanted modes, as discussed in references [10,11]. Light can be confined inside the hollow core of the HC-ARF via the mechanism of antiresonant effect [12], and strengthened by inhibited coupling between core and cladding modes [13]. In general, the fundamental mode transmission without higher-order modes (HOMs) in HC-ARFs is required in some applications such as high-power pulsed laser delivery [14], data transmission [15], and biochemical sensing [16]. Several recent works were reported to suppress HOMs in HC-ARFs through the resonant coupling between HOMs in the core and tube modes in the cladding based on the structure parameter optimization to achieve only single-mode operation [17,18,19].

In contrast to single-mode fibers (SMFs), few-mode fibers allow both the fundamental mode and a few HOMs to be guided, promoting applications in few-mode fiber amplifiers [20], mode-division-multiplexed systems [21], and multi-parameter sensing [22] with attractive features that SMFs cannot implement. Furthermore, only HOM propagation is preferred in a variety of applications instead of the fundamental mode because of its complex intensity pattern, phase property, and polarization distribution. The advantageous applications of HOMs include all-fiber Raman probe [23], ultra-large effective area high-power lasers [24], and nonlinear optics [25]. In particular, the second-order modes, including four degenerate vector modes, TE_01_ (azimuthally polarized), TM_01_ (radially polarized), HE_21_(even), and HE_21_(odd) modes, possess doughnut-shaped field distribution with unique polarization [26]; such properties of the second-order mode provide opportunities in many significant applications such as high-resolution microscopy [27], material micromachining [28], particle trapping and optical tweezers [29], mode division multiplexing systems [30], plasmonic focusing [31], and so forth [32]. Additionally, the second-order mode in few-mode fibers could be served as exciting light to realize all-fiber sensors with simple structures [33].

Propagation properties of only second-order modes with suppression of the fundamental mode were investigated in different types of microstructure optical fibers. In solid-core bandgap photonic crystal fibers (PCFs), the transmission properties of only the LP_11_ mode at the red transmission band edges were investigated [34]. Recently, a new design of the solid-core PCF was proposed to suppress the fundamental mode by the coupling between the fundamental mode and the cladding mode to keep the second-order modes with low confinement loss [35]. Another theoretical work by Chen et al. focused on selectively eliminating the fundamental mode or the second-order mode through the use of complex structures with a high-index core and a photonic bandgap cladding [36]. The hollow-core fibers (HCFs) have unique advantages for ultra-short pulse laser delivery over solid-core fibers, and the ability to guide only second-order vector mode in the HCFs will open up many new applications. In the hollow-core bandgap PCFs, Digonnet et al. claimed that the guided core modes are determined by the frequencies, and only HOMs can be guided in the core due to the cutoff of the fundamental mode at the lower-frequency band edge [37]. Moreover, the favor propagation of the LP_11_ mode was presented in inhibited-coupling guiding tubular fibers with modified cladding structure [38]. However, the design for second-order mode operation they investigated involved degenerated modes (LP_11_ mode group) instead of separated vector modes (TE_01,_ TM_01_, HE_21_(even), and HE_21_(odd) modes) in these fibers, which may be not ideal for many applications in which a pure second-order vector mode is desirable, such as high-resolution microscopy [27], material micromachining [28], particle trapping and optical tweezers [29], and so forth.

In this paper, we firstly demonstrate the unique modal-guiding property in a HC-ARF, where only one second-order vector mode is well guided with the suppression of the fundamental mode and other second-order vector modes at the red side of the transmission window. We further exploit the detailed characteristics of the HC-ARF in terms of dispersions of core modes and cladding modes, confinement losses of core modes, mode patterns, and the coupling mechanism at the transmission red edge. By understanding the coupling mechanism, we propose that there are specific wavelength ranges in which only one second-order vector mode of TE_01_, TM_01_, or HE_21_ mode can be well guided with suitable design of fiber structure parameters. Through the modal engineering by optimizing the fiber structure, only one second-order vector mode can be guided in a HCF. Since the HCFs are extremely important for ultra-short pulse laser delivery, our selective second-order vector mode guiding in a HC-ARF will open up advanced applications such as laser drilling [39], particle trapping [40], and microscopy [41] by using ultra-short pulse lasers.

## 2. Fiber Structures and Modal Engineering Conception

**Figure 1:**
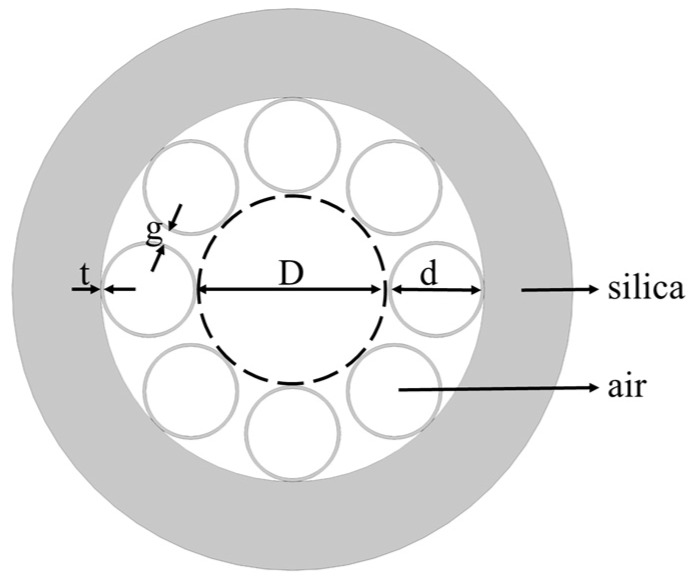
Cross-section of the proposed hollow-core antiresonant fiber (HC-ARF).

Here, a simple HC-ARF structure consisting of a hollow-core surrounded by eight identical silica tubes (Figure 1) is used to investigate the modal characteristics. The core diameter of *D*, the inner tube diameter of *d*, the tube thickness of *t*, and the tube gap of *g* are related by the expression *D* = (*d* + 2*t* + *g*)/sin(π/8) − (*d* + 2*t*) [42]. Modal characteristics are calculated using a full-vector finite element method (FEM) with the initial parameters of *D* of 41 μm, *t* of 0.394 μm, *g* of 3 μm, and *d* of 19.769 μm. The white region represents the air with the refractive index of 1. The gray region represents the background material of silica. The dispersion of silica can be calculated using the Sellmeier equation [43] as follows:

(1) $$n(\lambda )=\sqrt{1+\frac{B_{1}\lambda^{2}}{\lambda^{2}-\lambda_{1}^{2}}+\frac{B_{2}\lambda^{2}}{\lambda^{2}-\lambda_{2}^{2}}+\frac{B_{3}\lambda^{2}}{\lambda^{2}-\lambda_{3}^{2}}},$$

where *B*_1_ = 0.6961663, *B*_2_ = 0.4079426, *B*_3_ = 0.8974794, *λ*_1_ = 0.0684043 µm, *λ*_2_ = 0.1162414 µm, and *λ*_3_ = 9.896161 µm [43]. The material loss is neglected, since the material absorption of silica is quite low within the explored wavelength range [3,9,17,19,35].

The FEM is utilized to investigate the mode characteristics and propagation constants of the core modes and cladding modes. The confinement loss is calculated through the use of a perfectly matched layer (PML) to surround the simulation area as the imaginary part of the eigenvalue returned by the modal solver [3]. The mesh size is carefully optimized to make the calculation accurate. In this case, extremely fine meshes, with element sizes of less than *λ*/5 in air and less than *λ*/6 in the thin glass tube regions, are needed to achieve accurate results [3]. Additionally, the dispersion curves of core modes and cladding modes are depicted with the real part of the mode effective refractive index. The mode confinement loss can be calculated by the equation in reference [44] which is related to the imaginary part of the mode effective refractive index. 

**Figure 2:**
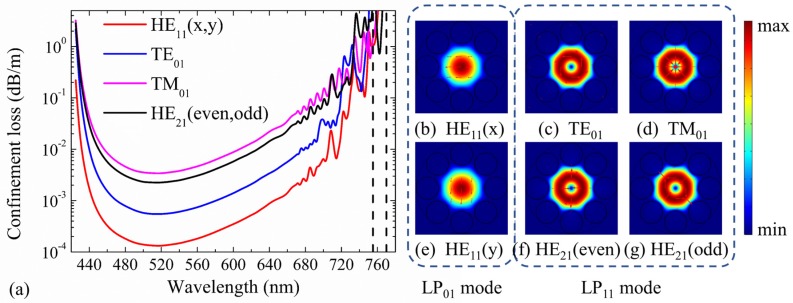
(**a**) The confinement loss curves, and (**b**–**g**) electric field distributions of HE_11_(*x*, *y*), TE_01_, TM_01_, and HE_21_(even, odd) core modes in the HC-ARF at the wavelength of 570 nm. The fiber structure parameters are *D* = 41 μm, *t* = 0.394 μm, *g* = 3 μm, *d* = 19.769 μm, and *d*/*D* = 0.482. Both HE_11_(*x*, *y*) and HE_21_(even, odd) are degenerated modes in two different polarization directions.

Figure 2a shows the spectra of confinement losses of the fundamental modes (HE_11_(*x*, *y*)) and second-order mode group (TE_01_, TM_01_, and HE_21_(even, odd)) as a function of operating wavelength in the second transmission band. Moreover, the electric field distributions of these core modes are illustrated in Figure 2b–g, where the arrows indicate the polarization directions. Within the working wavelength range of 420–780 nm, the spectrum exhibits a bandwidth of over 300 nm, and the band edges of this transmission window are around 420 nm and 770 nm, respectively. 

As shown in Figure 2a, the confinement losses of second-order modes are much larger than those of the fundamental modes at the center part of transmission band. However, the results are quite different at the red side of the transmission window. As the wavelength is larger than 670 nm, the losses of HE_11_, TE_01_, TM_01_, and HE_21_ modes all fluctuate with the wavelength, which can be explained by the fact that the core modes couple to cladding modes localized in silica tubes [45]. The similar loss fluctuations at the red side of the second transmission band are also observed in the HC-ARF with seven tubes [45]. Although the losses of the different modes increase with fluctuations at the red side of the band, the loss of the fundamental mode is always lower than those of HOMs due to the strong coupling between HOMs and tube modes with optimized *d*/*D* of 0.7 [45]. However, some interesting phenomena occur in our simulation, which are quite different from the results in reference [45]. For example, the HE_21_ mode is well guided with the lowest confinement loss of 0.72 dB/m at 762.5 nm, and the losses of the fundamental mode and other HOMs are much higher at this wavelength. The modal properties within the wavelength range from 755 nm to 770 nm, marked with dashed line in Figure 2a, are further investigated in detail in Section 3.1. The cladding modes are affected by the tube structure parameters, which can be optimized to produce the desirable coupled cladding modes. Thus, they will have mode resonance with unwanted core modes, filtering specific core modes even it is a fundamental mode. Through the proposed modal engineering, only second-order vector mode operation can be implemented.

## 3. Characteristics of Second-Order Modes in HC-ARFs and Discussion

### 3.1. Characteristics of Only HE_21_ Mode Propagation

**Figure 3:**
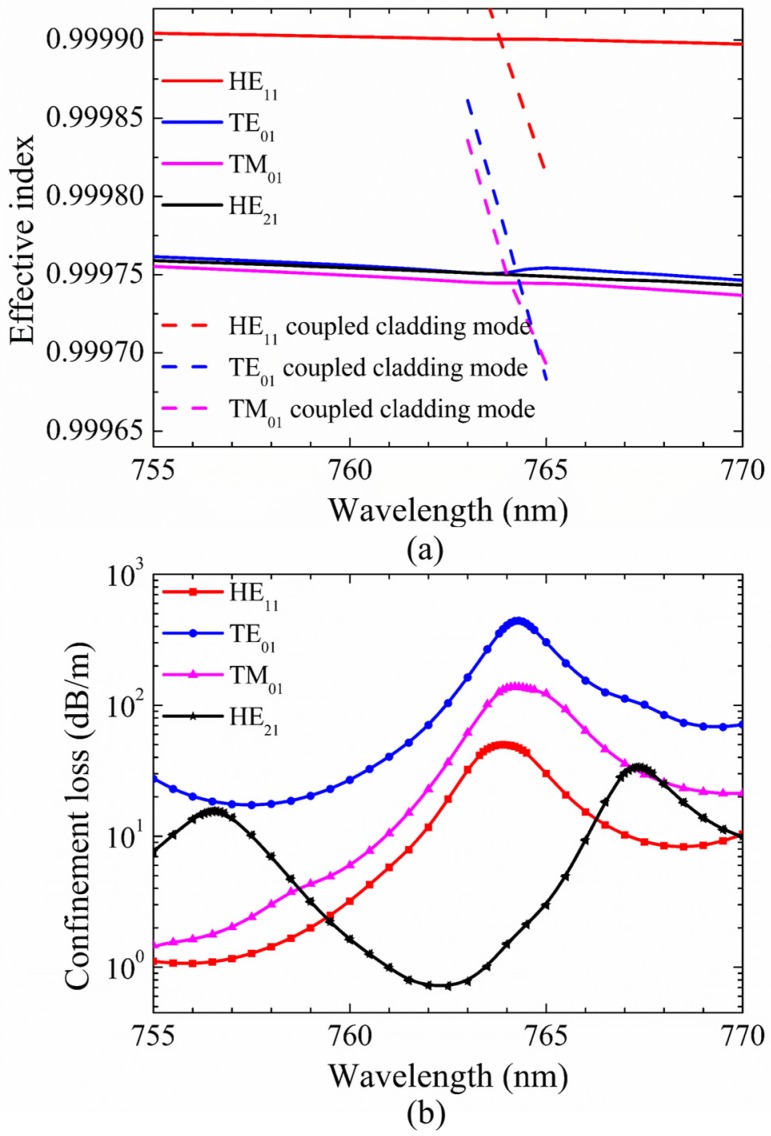
(**a**) The dispersion curves of HE_11_, TE_01_, TM_01_, and HE_21_ core modes and related coupled cladding modes. (**b**) Confinement losses of different core modes. The fiber structure parameters are *D* = 41μm, *t* = 0.394 μm, *g* = 3 μm, *d* = 19.769 μm, and *d*/*D* = 0.482. The step of calculated points is 0.5 nm and the step is added to 0.1 nm at the peak region of curves.

**Figure 4:**
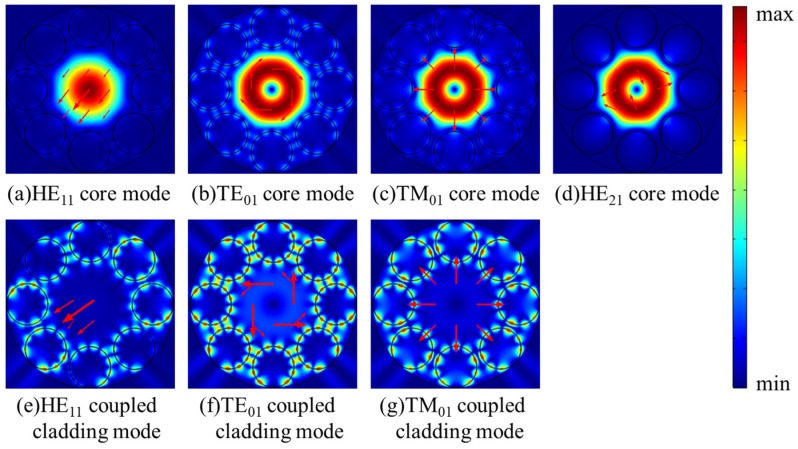
(**a**–**g**) The electric field distributions of the core modes and related coupled cladding modes at 764 nm.

In order to gain more detailed insight into the fluctuation in Figure 2a, the dispersion curves and confinement loss curves of HE_11_, TE_01_, TM_01_, and HE_21_ modes at the wavelength range of 755–770 nm are shown in Figure 3a,b. The step of calculated points is 0.5 nm and it is added to 0.1 nm at the peak region of the curves. In Figure 3b, the confinement losses of HE_11_, TE_01_, and TM_01_ core modes are larger than that of the HE_21_ mode at the wavelength range from 759.5 nm to 766.0 nm. Furthermore, the losses of HE_11_, TE_01_, and TM_01_ core modes reach to high peaks at the wavelength around 764 nm. To explain the high loss peaks of core modes around 764 nm, the electric field distributions of HE_11_, TE_01_, TM_01_, and HE_21_ core modes and the related cladding modes at 764 nm are illustrated in Figure 4a–g. Additionally, the dispersion curves of the core modes and the related cladding modes are plotted in Figure 3a to further explain these high loss peaks.

As shown in Figure 3a, the intersection points between the dispersion curves of HE_11_, TE_01_, and TM_01_ core modes and those of related coupled cladding modes correspond to phase matching conditions, leading to high loss peaks of HE_11_, TE_01_, and TM_01_ core modes around 764 nm. It should be noted that, although the dispersion curves of TE_01_ and TM_01_ coupled cladding modes cross with that of the HE_21_ mode around 764 nm, the loss of HE_21_ mode is kept with the low value without resonant coupling due to the mismatched polarization directions. Furthermore, the electric field distributions in Figure 4a–g show that the HE_11_, TE_01_, and TM_01_ core modes can couple with related cladding modes with matched polarization directions at 764 nm, while the HE_21_ mode can be well confined in the core due to the mismatched polarization directions at 764 nm. In other words, the high loss peaks of HE_11_, TE_01_, and TM_01_ core modes around 764 nm arise from the resonance coupling between core modes and related cladding modes as the effective index and polarization matching conditions are simultaneously satisfied. 

Mode extinction ratio (MER) is commonly defined as the loss ratio between different modes to quantify the degree of mode suppression; here, we introduce MER of the fundamental mode and other second-order mode to the desirable specific second-order vector mode, describing the propagation performance of desirable second-order vector mode. In Figure 3b, the loss of HE_21_ mode is 1.02 dB/m, and the losses of HE_11_, TE_01_, and TM_01_ modes are 46.28 dB/m, 267.57 dB/m, and 101.76 dB/m at 763.5 nm, respectively. Thus, the MER for HE_21_ mode is larger than 45 at 763.5 nm; in this case, only one second-order vector mode, HE_21_ mode, is well guided at the specific wavelength. We also noticed that this remarkable MER for the second-order vector mode cannot be observed in the first transmission band due to the relatively weak coupling from the cladding modes. Additionally, similar results of only HE_21_ mode guiding can be obtained around 413.5 nm at the red side of the third transmission band, which verify the reliability of the unique modal-guiding property at the red side of transmission bands. 

### 3.2. Characteristics of Only TM_01_ Mode Propagation

Based on our understanding of only HE_21_ mode guiding with the suppression of the fundamental mode and other second-order vector modes at the red side of the transmission window, we also realize the propagation of only TM_01_ or TE_01_ mode with suppression of other modes by choosing suitable parameters of HC-ARFs. To keep the invariance of the transmission window, the initial parameters of core diameter and tube thickness are always kept constant as *D* of 41 μm and *t* of 0.394 μm, and the dispersion and confinement loss are also investigated in the red side of the transmission window. 

**Figure 5:**
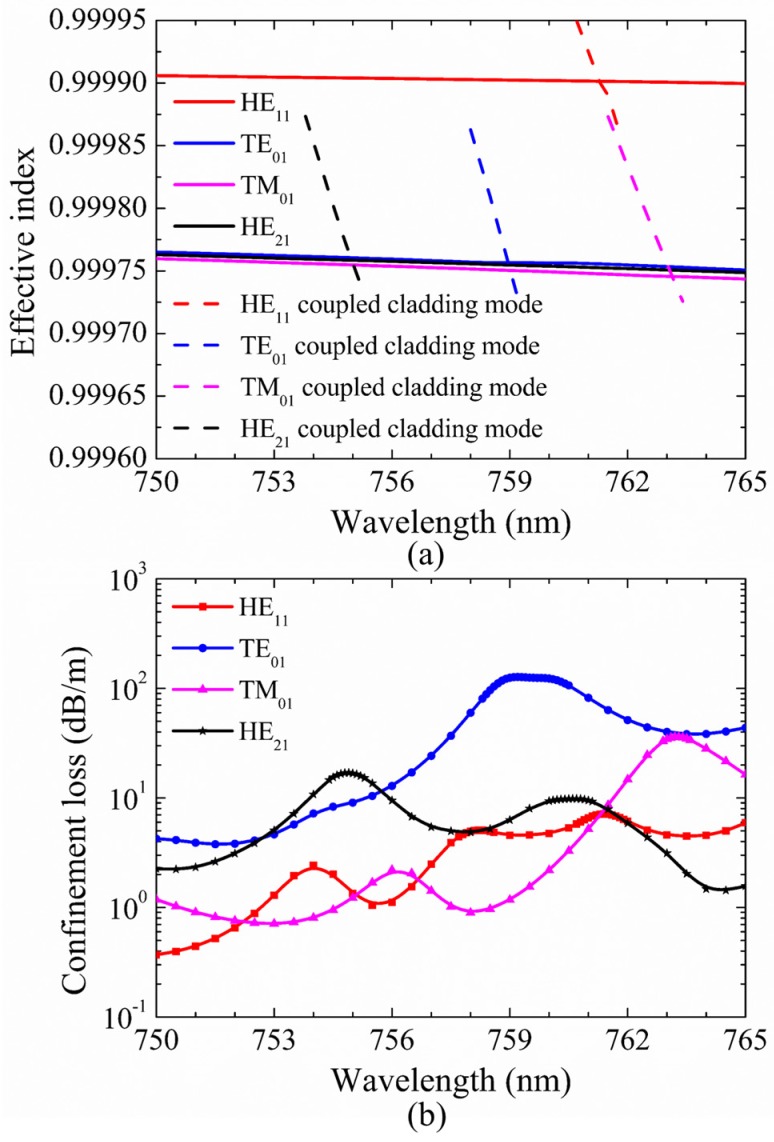
(**a**) The dispersion curves of HE_11_, TE_01_, TM_01_, and HE_21_ core modes and related cladding modes. (**b**) Confinement losses of different core modes. The fiber structure parameters are *D* = 41 μm, *t* = 0.394 μm, *g* = 2 μm, and *d* = 21.389 μm, and *d*/*D* = 0.522.

**Figure 6:**
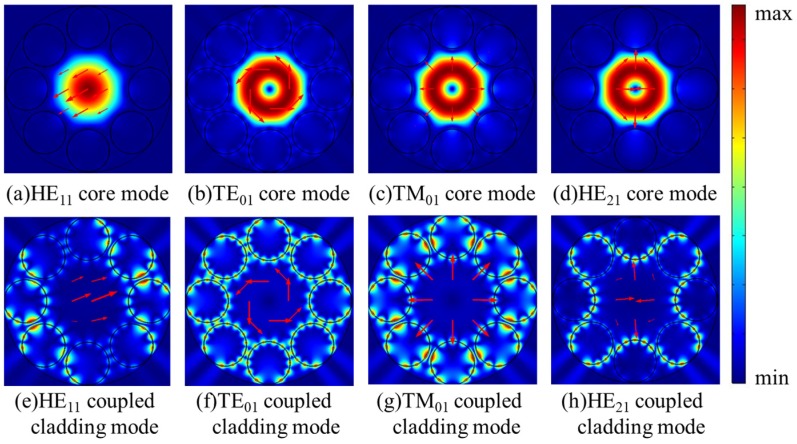
(**a**–**h**) The electric field distributions of the core modes and related cladding modes at the wavelengths corresponding to relatively high loss peaks: (**a**,**e**) at the wavelength of 761.4 nm; (**b**,**f**) at the wavelength of 759.2 nm; (**c**,**g**) at the wavelength of 763.3 nm; (**d**,**h**) at the wavelength of 754.9 nm.

In this part, we propose the propagation of only TM_01_ mode with low confinement loss. The fiber structure parameters are *D* = 41 μm, *t* = 0.394 μm, *g* = 2 μm, and *d* = 21.389 μm. Figure 5a depicts the dispersion curves of HE_11_, TE_01_, TM_01_, and HE_21_ core modes and related coupled cladding modes. In order to make the Figure 5a clear, only the dispersion curves of cladding modes corresponding to relatively high loss peaks of the core modes are depicted. Figure 5b shows the confinement losses of different core modes at the wavelength range from 750 nm to 765 nm. Several confinement loss peaks of HE_11_, TM_01_, and HE_21_ core modes occur at the wavelength range from 750 nm to 765 nm. The electric field distributions of the core modes and related coupled cladding modes at particular resonant wavelengths are shown in Figure 6a–h. 

**Figure 7:**
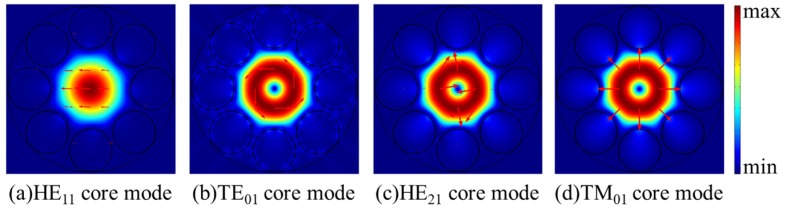
(**a–d**) The electric field distributions of the core modes at 758 nm.

As shown in Figure 5, the confinement losses of HE_11_, TE_01_, TM_01_, and HE_21_ core modes rise to relatively high loss peaks when the dispersions curves of HE_11_, TE_01_, TM_01_, and HE_21_ core modes and related cladding modes are crossed at particular wavelengths. The wavelengths corresponding to the relatively high loss peaks of HE_21,_ TE_01_, HE_11_, and TM_01_ modes are 754.9 nm, 759.2 nm, 761.4 nm, and 763.3 nm, respectively. At these wavelengths, the HE_11_, TE_01_, TM_01_, and HE_21_ core modes couple with cladding modes, as shown in Figure 6a–h. The TM_01_ mode is well guided with the low losses of 0.80 dB/m at 754 nm and 0.90 dB/m at 758 nm. The confinement losses of HE_11_, HE_21_, and TE_01_ modes are 4.95 dB/m, 4.86 dB/m, and 59.82 dB/m, respectively. Thus, the MER for the TM_01_ mode is larger than 5 at 758 nm. Additionally, the electric field distributions of HE_11_, TE_01_, TM_01_, and HE_21_ core modes at 758 nm are shown in Figure 7a–d. The electric fields of HE_11_, TE_01_, and HE_21_ core modes are partly distributed into the cladding tubes, indicating the resonant coupling loss, while the TM_01_ mode can be well confined in the hollow core. The propagation of TM_01_ mode with low confinement loss appears at two wavelength ranges around 754 nm and 758 nm with the proposed fiber structure parameters.

### 3.3. Characteristics of Only TE_01_ Mode Propagation

**Figure 8:**
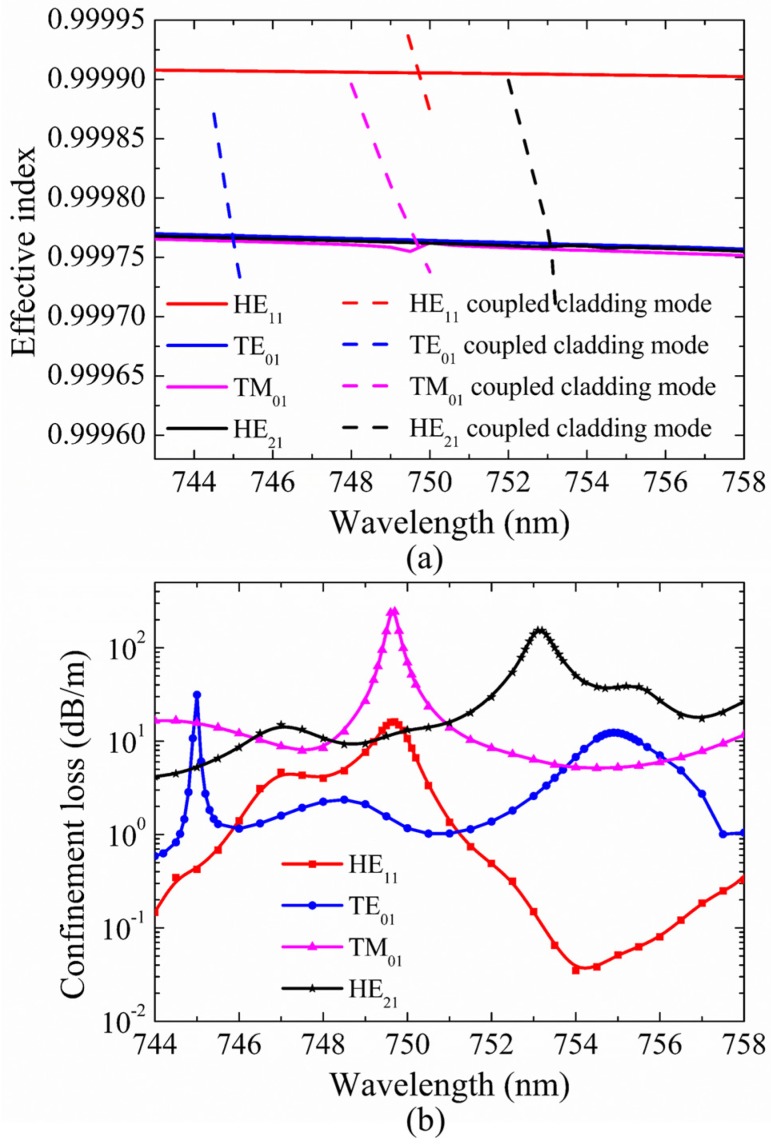
(**a**) The dispersion curves of HE_11_, TE_01_, TM_01_, and HE_21_ core modes and related cladding modes. (**b**) Confinement losses of different core modes. The fiber structure parameters are *D* = 41 μm, *t* = 0.394 μm, *g* = 0 μm, *d* = 24.628 μm, and *d*/*D* = 0.601.

**Figure 9:**
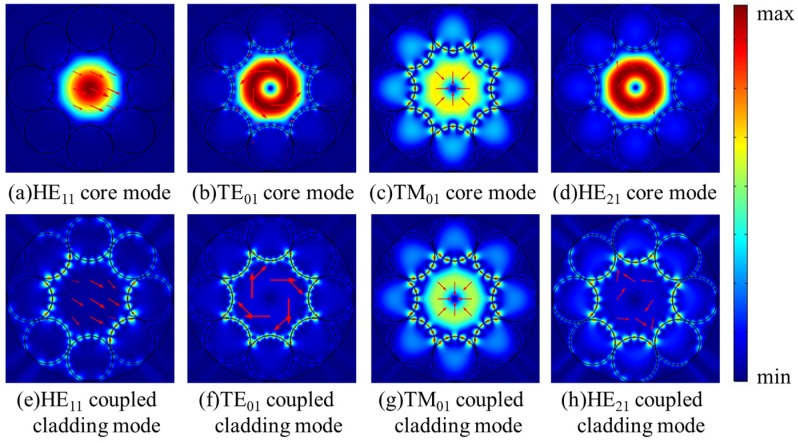
(**a**–**h**) The electric field distributions of the core modes and related cladding modes at the wavelengths corresponding to relatively high loss peaks: (**a**,**e**) at the wavelength of 749.7 nm; (**b**,**f**) at the wavelength of 745.0 nm; (**c**,**g**) at the wavelength of 749.7 nm; (**d**,**h**) at the wavelength of 753.1 nm.

So far, the propagation of only TM_01_ and only HE_21_ modes with low confinement loss were discussed in HC-ARFs with different tube gaps. In this part, we set tube gap *g* as 0 μm and tube diameter *d* as 24.628 μm to propose only TE_01_ mode propagation with the suppression of the fundamental mode and other second-order vector modes. Figure 8 presents the dispersion and confinement loss curves of HE_11_, TE_01_, TM_01_, and HE_21_ core modes at the wavelength range from 744 nm to 758 nm. In Figure 8b, the losses of HE_11_, TE_01_, TM_01_, and HE_21_ core modes all fluctuate within the investigated wavelength range and the TE_01_ mode possesses high MER around 750 nm. Similarly, only the dispersion curves of the cladding modes corresponding to relatively high loss peaks are depicted in Figure 8a to make the figure clear. Moreover, the electric field distributions of the core modes and the coupled cladding modes at the wavelengths corresponding to relatively high loss peaks are shown in Figure 9a–h.

**Figure 10:**
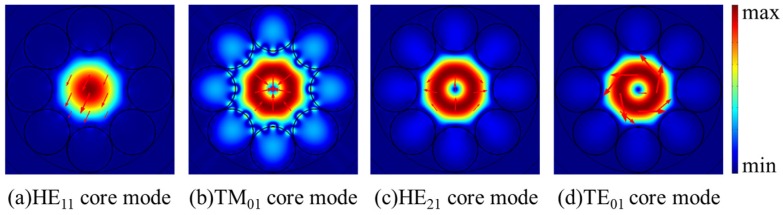
(**a–d**) The electric field distributions of the core modes at 750 nm.

As shown in Figure 8a, the dispersion curves of HE_11_, TE_01_, TM_01_, and HE_21_ core modes cross with those of related cladding modes at particular wavelengths, where the losses of HE_11_, TE_01_, TM_01_, and HE_21_ core modes reach to high peaks in Figure 8b. The loss peaks of TE_01_, HE_11_, TM_01_, and HE_21_ modes reach to 31.42 dB/m, 16.11 dB/m, 243.66 dB/m, and 153.28 dB/m at 745.0 nm, 749.7 nm, 749.7 nm, and 753.1 nm, respectively. At these wavelengths, the core modes couple to cladding modes residing in silica tubes as shown in Figure 9a–h. In other words, the core modes couple to related cladding modes as the effective index and polarization matching conditions are both satisfied, causing high confinement loss at resonant wavelengths, which is the same mechanism as the results demonstrated in the above sections. Based on this coupling mechanism, HE_11_ and TM_01_ core modes couple to the related cladding modes and reach to high loss peaks around 750 nm. Although the HE_21_ core mode does not couple with the related cladding mode around 750 nm, the HE_21_ core mode keeps with relatively high confinement loss within the studied wavelength range. The confinement losses of HE_11_, HE_21_, and TM_01_ modes are 10.65 dB/m, 13.20 dB/m, and 69.61 dB/m, respectively, which are much larger than the confinement loss of 1.17 dB/m of the TE_01_ mode at 750 nm, and the MER for the TE_01_ mode is larger than 9 at 750 nm. Moreover, the electric field patterns of the core modes in Figure 10a–d show that HE_11_, TM_01_, and HE_21_ modes are distributed in the cladding tubes, while the TE_01_ mode is well confined in the hollow core without of the resonance coupling with cladding modes. Therefore HE_11_, HE_21_, and TM_01_ modes can be effectively suppressed and the TE_01_ mode can be well guided with relatively low transmission loss with the proposed fiber structure parameters. 

We also have a simple investigation checking the sensitivity of the optical properties to small variations of 1% increase or decrease in *d*/*D* due to the fabrication tolerance of the HC-ARF. For example, the optimized MER for the HE_21_ mode is 46.75 at 763 nm and 42.63 at 763.8 nm with −1% and +1% changes of *d*/*D* of 0.482; it is not highly sensitive to the small variation of the designed parameters. We noticed it is quite similar for the TM_01_ and TE_01_ modes for small variation, where the slight change of *d*/*D* leads to only a very small shift of the wavelengths corresponding to the optimized MER at different *d*/*D*. Indeed, the phenomenon of propagation of second-order vector mode with the lowest confinement loss can be observed for the slight change of *d*/*D*, and optimized MERs and working wavelengths are similar, which indicate that the stable propagation of second-order vector mode in HC-ARFs can be achieved even with the slight variation of fabrication parameters.

As we mentioned, the previous reports about the mode propagation in HC-ARFs were mainly about fundamental mode with suppression of HOMs based on the mode coupling between core HOMs and tube modes, which was studied comprehensively. However, only HOM propagation in HC-ARFs was rarely investigated. Considering the wide applications of only one second-order vector mode, our research about propagation of only one second-order vector mode with suppression of the fundamental mode in the HC-ARF is significantly important. Actually, it is much more difficult to suppress the fundamental mode while keeping the second-order mode with high MER because the fundamental mode generally has lowest confinement loss. In this section, we proposed the propagation of only TE_01_, TM_01_, or HE_21_ modes with low confinement loss at specific wavelengths in HC-ARFs, and also investigated their modal characteristics, confinement losses, and mechanism of mode coupling in detail. To the best of our knowledge, this is the first discussion about propagation of only one second-order vector mode with low confinement loss in HC-ARFs. It should be noted that the MER for the second-order mode is not fully optimized since we keep the core diameter *D* and the tube thickness *t* constant and only modify the tube gap *g* to vary the coupled cladding mode. We believe that much larger MER for second-order mode can be achieved based on further optimized structures in our future work.

## 4. Conclusions

We firstly demonstrated the unique modal-guiding property in a HC-ARF, whereby only a second-order vector mode can be well guided with low confinement loss while the fundamental mode and other second-order vector modes reach to high loss peaks at the red side of transmission band. The specific modal characteristics can be interpreted by the mode coupling between the core modes and cladding modes when phase matching conditions are satisfied in terms of effective refractive index and polarization matching. The second-order vector mode characteristics including dispersion properties, confinement losses, and mode patterns at the red edge of transmission windows were investigated, and only one second-order vector mode guiding of TE_01_, TM_01_, or HE_21_ modes is theoretically implemented at specific wavelength ranges with suppression of the fundamental mode in the HC-ARF. It is expected that the propagation of only TE_01_, TM_01_, or HE_21_ modes with low confinement loss in HC-ARFs will have fruitful applications for ultra-short pulse laser delivery, including femtosecond laser drilling, particle trapping, and microscopy, by using our proposed design and strategies.
